# Exploring the Volatility, Phase Transitions, and Solubility Properties of Five Halogenated Benzaldehydes

**DOI:** 10.3390/molecules30071551

**Published:** 2025-03-31

**Authors:** Ana R. R. P. Almeida, Bruno D. A. Pinheiro, Gastón P. León, Bogdan Postolnyi, João P. Araújo, Manuel J. S. Monte

**Affiliations:** 1 Research Centre in Chemistry (CIQUP), Institute of Molecular Sciences (IMS), Department of Chemistry and Biochemistry (DQB), Faculty of Sciences, University of Porto, Rua do Campo Alegre 687, 4169-007 Porto, Portugal; brunodapinheiro@gmail.com (B.D.A.P.); gaston.leon@fc.up.pt (G.P.L.); mjmonte@fc.up.pt (M.J.S.M.); 2 Institute of Physics for Advanced Materials, Nanotechnology and Photonics (IFIMUP), Department of Physics and Astronomy (DFA), Faculty of Sciences, University of Porto, Rua do Campo Alegre 687, 4169-007 Porto, Portugal; b.postolnyi@fc.up.pt (B.P.); jearaujo@fc.up.pt (J.P.A.)

**Keywords:** 4-chlorobenzaldehyde, 4-bromobenzaldehyde, 2,3-dichlorobenzaldehyde, 2,4-dichlorobenzaldehyde, 2,6-dichlorobenzaldehyde, volatility, phase transitions, solubility

## Abstract

Halogenated benzaldehydes possess unique chemical properties that render them valuable in pharmaceutical synthesis, pesticide formulation, and dye production. However, thorough thermodynamic data for these compounds remain scarce. This study aims to fill this knowledge gap by investigating key physical properties of several halogenated benzaldehydes, namely 4-chlorobenzaldehyde, 4-bromobenzaldehyde, 2,3-dichlorobenzaldehyde, 2,4-dichlorobenzaldehyde, and 2,6-dichlorobenzaldehyde. The physical properties determined in this study include volatility, phase transitions, and water solubility, all of which are crucial for predicting the environmental fate of these compounds. The vapor pressures of both crystalline and liquid phases were measured using a reliable static method, allowing for the determination of standard molar enthalpies, entropies, and Gibbs energies of sublimation and vaporization, as well as their triple points. The melting temperature and molar enthalpy, along with the isobaric molar heat capacity of the crystalline phase, were assessed using differential scanning calorimetry. Water solubility was evaluated at 25 °C through the saturation shake-flask method, complemented by ultra-violet visible spectroscopy. By combining sublimation and solubility data, additional properties such as Gibbs energies of hydration and Henry’s law constants were derived. The experimental results were integrated into existing databases, enhancing the predictive models for properties including melting temperature, vapor pressure, solubility, Gibbs energy of hydration, and Henry’s constant. These findings significantly improve the environmental modeling capabilities, providing valuable insights into the mobility and fate of halogenated benzaldehydes in various environmental contexts.

## 1. Introduction

Halogenated benzaldehydes are derivatives of benzaldehyde in which one or more hydrogen atoms on the benzene ring are substituted with halogen atoms (fluorine, chlorine, bromine, or iodine). These substitutions significantly influence the compounds’ chemical and physical properties, making them versatile intermediates across various industries. The increased reactivity introduced by the halogen substituents proves that these compounds are valuable for synthesizing drugs, active pharmaceutical ingredients (APIs) [1,2,3,4], pesticides [5,6,7], dyes, and pigments [7,8]. Furthermore, their capacity for further chemical transformations underscores their importance in a range of applications, including inhibiting mild steel corrosion in hydrochloric acid solutions or serving as guest molecules in the formation of inclusion complexes with cyclodextrins [9,10,11]. Despite their extensive applications, there remains a notable lack of fundamental data on their physicochemical properties, which hampers the full understanding of their behavior and environmental impact.

**Figure 1:**
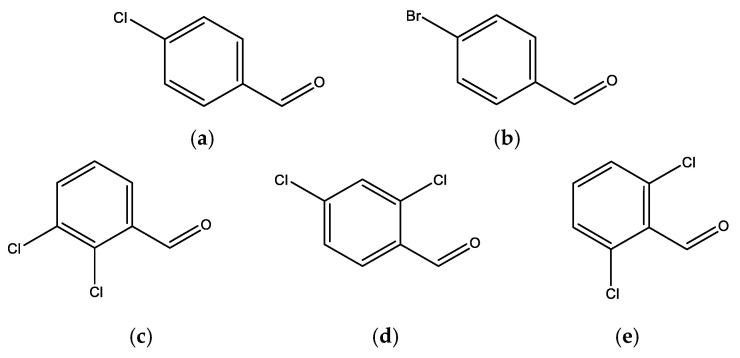
Structural formulae of the halogenated benzaldehydes studied in this work: (**a**) 4-Chlorobenzaldehyde, (**b**) 4-Bromobenzaldehyde, (**c**) 2,3-Dichlorobenzaldehyde, (**d**) 2,4-Dichlorobenzaldehyde and (**e**) 2,6-Dichlorobenzaldehyde.

This study centers on the experimental determination and estimation of key properties of the five halogenated benzaldehydes, which are schematically represented in Figure 1: 4-chlorobenzaldehyde, 4-bromobenzaldehyde, 2,3-dichlorobenzaldehyde, 2,4-dichlorobenzaldehyde, and 2,6-dichlorobenzaldehyde. Specifically, this work analyses their volatility, phase transitions, and water solubility, as these properties influence their environmental fate and transport. For instance, a higher volatility increases the likelihood of atmospheric loss, allowing these compounds to be transported over long distances before deposition, which can potentially contribute to surface water pollution. In contrast, water solubility is crucial for understanding how these compounds interact with ecosystems; highly soluble substances are more likely to undergo biodegradation but are less prone to accumulate in sediments or aquatic organisms [12].

By integrating experimental data on volatility and solubility, this study further evaluates properties such as Henry’s constant and Gibbs energy of hydration. Henry’s constant quantifies a compound’s partitioning between air and water, providing insights into its mobility and potential for contamination [13]. Gibbs energy of hydration, an essential thermodynamic parameter, quantifies the energy change that occurs when a compound transitions between gas and aqueous phases, shedding light on its solubility and stability in aquatic environments [14]. These properties are critical for predicting the mobility and environmental behavior of compounds and their potential impacts on water, and air systems.

In addition to expanding the experimental dataset, this work builds on earlier predictive models for benzene derivatives. Monte and Almeida [15] developed simple estimation equations for vapor pressures (or Gibbs energies) and enthalpies of sublimation based on the substituent groups present on a benzene ring [15]. Their approach, characterized by its ease of application and reliable accuracy (*σ* = 1.2 kJ·mol^−1^ for Gibbs energies and 2.4 kJ·mol^−1^ for enthalpies of sublimation), enabled predictions using a database of approximately 240 substituted benzenes. The prediction equations account for the number and contribution of each of the 30 substituent groups considered in those properties, the melting temperature of the substituted benzene, and the number and influence of interactions between substituting groups in *ortho*- and *para*- positions [15].

This method served as a foundation for exploring new approaches to predicting additional properties. Consequently, the dataset of the experimental Gibbs energies of sublimation was expanded to include values of vaporization, hydration, and solubility in water (at 25 °C), as well as additional melting data. With the exception of vaporization, it was concluded that the diversity of substituents considered did not allow the development of thorough methods to estimate properties related to hydration, solubility, and melting. As a result, the focus shifted to benzene derivatives with halogen atoms as substituents, encompassing around 40 compounds. In this sense, the same authors developed simple equations for estimating the properties of halogenated benzenes, which have significant applications in predicting the environmental mobility of these compounds, most of which are highly polluting. Properties considered included solubility in water, Henry’s constants, Gibbs energy of hydration, melting temperature, and vapor pressures (sublimation and vaporization) [16]. In both of the previous studies, no significant intramolecular interactions were found between halogen atoms [15,16] or between halogens and some other substituent groups. In this way, we planned to extend the estimation of these kinds of properties to other substituted benzenes, beginning with non-ionizable substituents that, like the halogen atoms, are not involved in relevant intramolecular interactions, such as the formyl group. By focusing on compounds with minimal intramolecular interactions, the research seeks to refine the predictive equations and contribute to a deeper understanding of the environmental mobility of these important chemicals.

The limited availability of reliable experimental data on the physicochemical properties of many organic compounds that impact the environment underscores the importance of these methods. This scarcity comes from several factors, including compound decomposition, the high costs associated with obtaining samples, and the challenges posed by the limitations of existing experimental methodologies.

## 2. Results

### 2.1. Volatility and Phase Transitions Study

**Figure 2:**
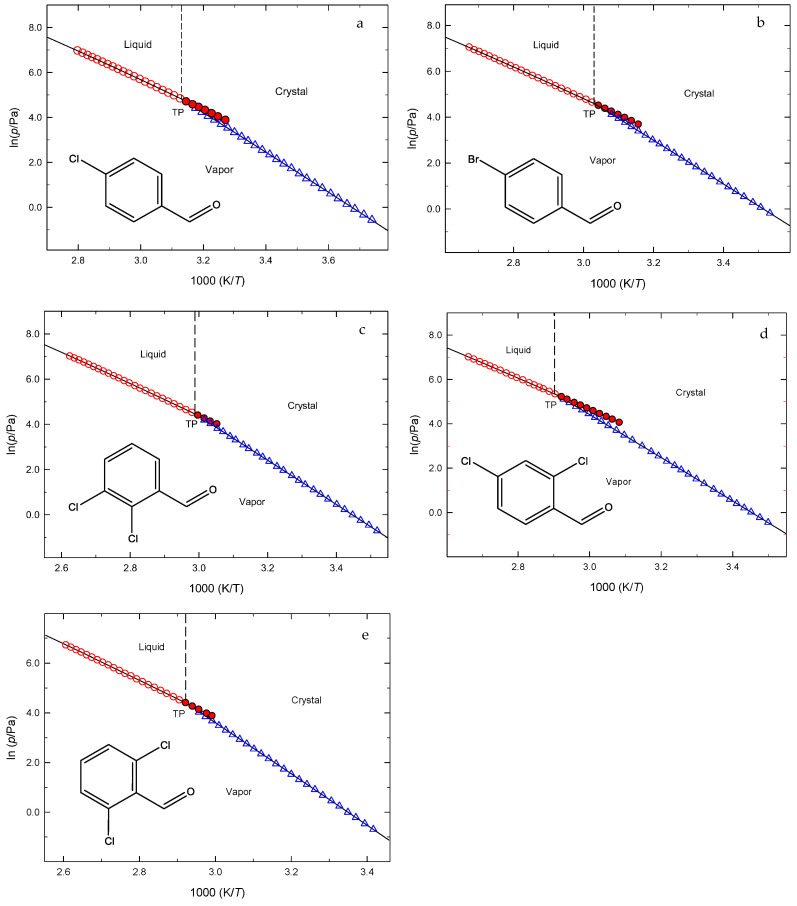
Phase diagrams in the vicinity of the triple points for the five compounds studied: **○**, vaporization; ●, vaporization (supercooled liquid); **Δ**, sublimation. TP: Triple point coordinates. (**a**) 4-Chlorobenzaldehyde: *T*_tp_ = 319.43 K; *p*_tp_ = 1.23 × 10^2^ Pa; (**b**) 4-Bromobenzaldehyde: *T*_tp_ = 330.02 K; *p*_tp_ = 99.1 Pa; (**c**) 2,3-Dichlorobenzaldehyde: *T*_tp_ = 334.82 K; *p*_tp_ = 87.6 Pa; (**d**) 2,4-Dichlorobenzaldehyde: *T*_tp_ = 343.90 K; *p*_tp_ = 2.01 × 10^2^ Pa; (**e**) 2,6-Dichlorobenzaldehyde: *T*_tp_ = 342.99 K; *p*_tp_ = 85.7 Pa.

Table 1 reports the vapor pressures of the crystalline and liquid (both stable and super-cooled) phases of the five compounds studied in this work. These results are also shown through phase diagrams in the vicinity of their triple points, represented in Figure 2. The experimental crystalline and liquid vapor pressures were independently fitted by the Clarke and Glew Equation (1) in its truncated form [17]:

(1) $$R\ln\left(\frac{p}{p^{{}^{\circ}}}\right)=-\frac{\Delta_{\mathrm{cd}}^{g}G_{m}^{{}^{\circ}}\left(\theta \right)}{\theta }+\Delta_{\mathrm{cd}}^{g}H_{m}^{{}^{\circ}}\left(\theta \right)\left(\frac{1}{\theta }-\frac{1}{T}\right)+\Delta_{\mathrm{cd}}^{g}C_{p,m}^{{}^{\circ}}\left(\theta \right)\left[\left(\frac{\theta }{T}\right)-1+\ln\left(\frac{T}{\theta }\right)\right]$$

where *R* is the molar gas constant, *p* is the vapor pressure at the temperature *T*, *p*° is a selected reference pressure (*p*° = 10^5^ Pa in this work), and *θ* is a reference temperature. The differences between gaseous and condensed phases of standard molar Gibbs energy, enthalpy, and the isobaric heat capacity are represented, respectively, by $\Delta_{\mathrm{cd}}^{g}G_{m}^{{}^{\circ}}$, $\Delta_{\mathrm{cd}}^{g}H_{m}^{{}^{\circ}}$, and $\Delta_{\mathrm{cd}}^{g}C_{p,m}^{{}^{\circ}}$. Table 2 lists the values of these properties for each compound at three distinct temperatures (*θ* = 298.15 K, *θ* = mean temperature of the experiments and *θ* = temperature of the triple point), and the uncertainties of $\Delta_{\mathrm{cd}}^{g}G_{m}^{{}^{\circ}}$ and $\Delta_{\mathrm{cd}}^{g}H_{m}^{{}^{\circ}}$, expressed as the expanded uncertainties (0.95 level of confidence, *k* = 2). Besides detailing these properties, this table also reports the values of $\Delta_{\mathrm{cd}}^{g}S_{m}^{{}^{\circ}}$, determined through the use of Equation (2), along with the corresponding uncertainties calculated by the Root Sum Square (RSS) method. The results of the vapor pressure calculations conducted using Equation (1) for those three temperatures are also presented in Table 2.

(2) $$\Delta_{\mathrm{cd}}^{g}S_{m}^{{}^{\circ}}(\theta )=\frac{\Delta_{\mathrm{cd}}^{g}H_{m}^{{}^{\circ}}(\theta )-\Delta_{\mathrm{cd}}^{g}G_{m}^{{}^{\circ}}(\theta )}{\theta }$$

The values of $\Delta_{\mathrm{cd}}^{g}C_{p,m}^{{}^{\circ}}$ are generally achieved only when accurate experimental data measured over ca. 50 K are reachable. The results of $\Delta_{\mathrm{cd}}^{g}C_{p,m}^{{}^{\circ}}$ (298.15 K) presented in Table 2, were derived directly from the least squares regression of the fittings of Equation (1) to the crystalline and to the liquid vapor pressure–temperature data (*R*^2^ = 1.0000 for all cases). For comparison reasons, the values of $\Delta_{\mathrm{cr}}^{g}C_{p,m}^{{}^{\circ}}$ were also calculated as $\Delta_{\mathrm{cr}}^{g}C_{p,m}^{{}^{\circ}}(\theta )=C_{p,m}^{{}^{\circ}}(g)-C_{p,m}^{{}^{\circ}}(cr)$, where the terms $C_{p,m}^{{}^{\circ}}(g)$ and $C_{p,m}^{{}^{\circ}}(cr)$ are, respectively, the gas and crystalline isobaric molar heat capacities. The results of $C_{p,m}^{{}^{\circ}}(g,298.15\;K)$ were calculated for the compounds studied through statistical thermodynamics using the Gaussian 09 software package [18], using the vibrational frequencies from G4 calculations. The crystalline molar heat capacities, $C_{p,m}^{{}^{\circ}}(cr)$, were determined at different temperatures using differential scanning calorimetry, enabling the fit to second-order polynomials in temperature, represented by Equation (3) and reported in Table 3. The correlation coefficients for all cases exceed 0.999. The values of $C_{p,m}^{{}^{\circ}}(cr,298.15\;K)$ were derived from these equations, allowing $\Delta_{cr}^{g}C_{p,m}^{{}^{\circ}}(298.15\;K)$ to be calculated. The gaseous and crystalline molar heat capacities determined in this work, the values estimated using the group contribution method proposed by Acree Jr. and Chickos [19], and the results of $\Delta_{cr}^{g}C_{p,m}^{{}^{\circ}}(298.15\;K)$ calculated in this study are reported in Table 4.

(3) $$C_{p,m}^{{}^{\circ}}(cr)/J\cdot K^{-1}\cdot {\mathrm{mol}}^{-1}=a+b(T/K)+(T^{2}/K)$$

The mean values of the melting properties, including the onset temperatures and enthalpies, determined in this study through DSC, closely match with those obtained using the static method, and are consistent with most results reported in the literature. All the results are reported in Table 5.

### 2.2. Solubility Measurements

**Figure 3:**
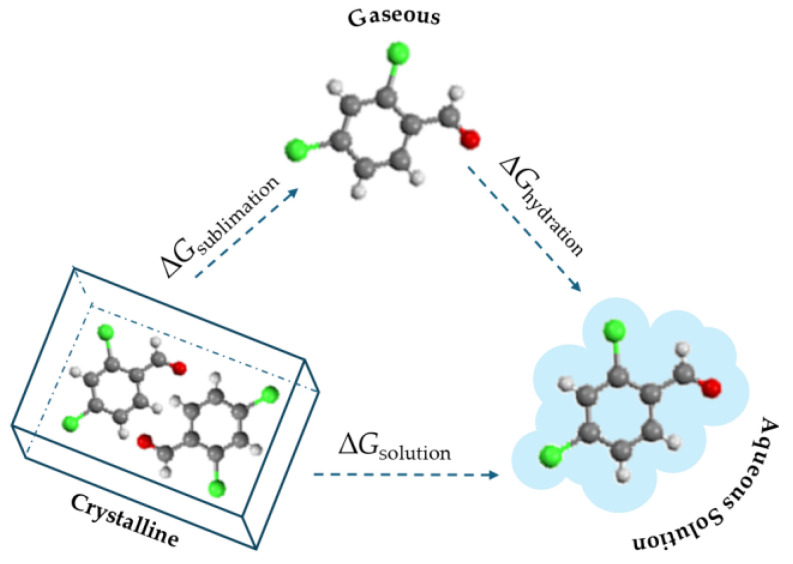
Thermodynamic cycle for transfer from crystal to gas and then to aqueous solution [28,29].

The equilibrium solubility of the five halogenated benzaldehydes were determined in water by saturation shake flask method, at atmospheric pressure, and at a temperature of 25 °C. The concentration was measured by absorbances at 260 nm for 4-chlorobenzaldehyde, 263 nm for 4-bromobenzaldehyde and 2,4-dichlorobenzaldehyde, 297 nm for 2,6-dichlorobenzaldehyde, and 306 nm for 2,3-dichlorobenzaldehyde, using a Cary-60 UV–vis (Agilent) spectrophotometer. X-ray diffraction (XRD) and differential scanning calorimetry of residual solids were used to identify the eventual phase transformations. The purified samples of each compound studied were used for all experimental determinations, including the solubility measurements. After reaching the equilibrium, the residual solid was dried and analyzed using both DSC and XRD. The melting properties obtained from DSC were compared with those of the original purified sample, following the same procedure for the XRD analysis. For all compounds, the melting properties and XRD patterns were similar, leading us to conclude that no polymorphs were present. As an example, the supporting information includes two thermograms and two XRD patterns for 2,4-dichlorobenzaldehyde (Figures S1 and S2). The pH of the saturated solutions was measured and is reported in Table S2. Three independent shake-flask experiments were conducted for each reported result listed in Table 6. The standard deviation of the mean result was also calculated. Considering the thermodynamic cycle schematically represented in Figure 3 [28,29] and Equation (4), the sublimation Gibbs energy and solubility results obtained in this study for the five substituted benzaldehydes were utilized to calculate the standard Gibbs energies of hydration. Equation (4) was derived from Equation (5), which is widely applicable to the solubility of solids [28,29].

(4) $$\Delta_{hydr}G_{m}^{{}^{\circ}}/\mathrm{kJ}\cdot {\mathrm{mol}}^{-1}=9.17-5.71\cdot \log_{10}(S_{\mathrm{cr}}/\mathrm{mol}\cdot m^{-3})-\Delta_{\mathrm{cr}}^{g}G_{m}^{{}^{\circ}}/\mathrm{kJ}\cdot {\mathrm{mol}}^{-1}$$

(5) $$S=\frac{p^{{}^{\circ}}}{RT}\exp\left(\frac{\Delta_{\mathrm{cr}}^{g}G_{m}^{{}^{\circ}}+\Delta_{hydr}G_{m}^{{}^{\circ}}}{-RT}\right)$$

The standard states in Equation (5) are *p*° = 1 × 10^5^ Pa for $\Delta_{\mathrm{cr}}^{g}G_{m}^{{}^{\circ}}$, *c*° = 1 mol·L^−1^ for $\Delta_{hydr}G_{m}^{{}^{\circ}}$ and *s*° = 1 mol·m^−3^ for solubility. Using the experimental results of $\Delta_{hydr}G_{m}^{{}^{\circ}}$, calculated through Equation (4), the values of Henry’s law volatility constants, $K_{H}^{pc}$ [30], were determined in the logarithm form by Equation (6), that was adapted from Modarressi et al. [31]. The results of these properties are reported in Table 6.

(6) $$\log_{10}(K_{H}^{pc}/\mathrm{kPa}\cdot m^{3}\cdot {\mathrm{mol}}^{-1})=\left(\frac{\Delta_{hydr}G_{m}^{{}^{\circ}}}{2.303\;RT}\right)+\log_{10}(RT/\mathrm{kJ}\cdot {\mathrm{mol}}^{-1})$$

## 3. Discussion

### 3.1. Vapor Pressures Study

To the best of our knowledge, no prior studies have reported the vapor pressures of the halogenated benzaldehydes presented in this work. Accurate measurements of vapor pressures are crucial to understand the behavior and environmental fate of organic compounds. These measurements are also crucial for regulatory agreement, assessing environmental impact, and determining the safe use of chemical substances [32].

According to Equation (7), the vapor pressure (which is related to volatility) of condensed compound (either crystalline or liquid), at a fixed temperature *T*, decreases as $\Delta_{\mathrm{cd}}^{g}G_{m}^{{}^{\circ}}(T)$ increases. At same time, Equation (2) shows that vapor pressure (and so volatility) decreases with the higher values of $\Delta_{\mathrm{cd}}^{g}H_{m}^{{}^{\circ}}(T)$ and with lower results of the term [$T\cdot \Delta_{\mathrm{cd}}^{g}S_{m}^{{}^{\circ}}(T)$]. The influence of these two contributions on the values of $\Delta_{\mathrm{cd}}^{g}G_{m}^{{}^{\circ}}(298.15\;K)$ of the compounds studied in this paper could be easily evaluated considering the thermodynamic properties, determined in this study and reported in Table 2. The significance of enthalpic effects on the phase stability of these chemicals is evident.

(7) $$\Delta_{\mathrm{cd}}^{g}G_{m}^{{}^{\circ}}(\theta )=-R\theta \ln\left(p/p^{{}^{\circ}}\right)$$

The sublimation vapor pressure values of the five halogenated benzaldehydes at *T* = 298.15 K, as summarized in Table 2, indicate their volatility ranking at this temperature as follows:

4-Chlorobenzaldehyde > 4-Bromobenzaldehyde > 2,4-Dichlorobenzaldehyde ~ 2,3-Dichlorobenzaldehyde > 2,6-Dichlorobenzaldehyde

The differences in vapor pressure results can be explained by examining the crystalline structures of these compounds [33,34,35], which provide information into the intermolecular interactions and structural effects influencing sublimation behavior and overall volatility.

For 4-chlorobenzaldehyde and 4-bromobenzaldehyde, the formyl groups are nearly coplanar with the phenyl rings. This planar arrangement facilitates moderate stabilization of the crystal lattice through C–H···O interactions, where the hydrogen atom of the formyl group in one molecule acts as a donor and the oxygen atom of another formyl group serves as an acceptor [33,34]. The higher vapor pressure value at 298.15 K of 4-chlorobenzaldehyde relative to 4-bromobenzaldehyde arises from its lower molecular weight and weaker intermolecular forces, making sublimation easier. The differences in the strength of the intermolecular interactions between the two compounds can be attributed to differences in the size, polarizability, and London dispersion forces of the chlorine and bromine atoms.

The three dichlorobenzaldehyde isomers exhibit unique molecular arrangements that affect their sublimation tendencies. While 2,3- and 2,6- dichlorobenzaldehydes are isostructural, their vapor pressure differences at the same temperature result from variations in intermolecular interaction energies [35]. In 2,3-dichlorobenzaldehyde, the chlorine atoms create weak and less stabilizing Cl···Cl and H···H contacts, resulting in lower lattice stability. In contrast, 2,6-dichlorobenzaldehyde exhibits stronger Cl···CHO interactions, with an energy contribution of −20.1 kJ·mol^−1^ compared to the weaker Cl···Cl interactions (−12.1 kJ·mol^−1^) in 2,3-dichlorobenzaldehyde, leading to lower values of vapor pressure or reduced volatility [35]. In 2,4-dichlorobenzaldehyde, weak C–H···O interactions involving the aldehyde oxygen atom and *ortho*-hydrogen atoms on the benzene ring stabilize the lattice. Additionally, π–π stacking interactions between benzene rings enhance stability. The combined effects result in vapor pressure values comparable to 2,3-dichlorobenzaldehyde. In the crystal structure of 2,6-dichlorobenzaldehyde, strong C=O···Cl interactions dominate, motivated by the polarity of the aldehyde group and chlorine substituents [35]. The combination of these strong and cohesive intermolecular forces results in sublimation being less favorable and reducing volatility for this compound.

### 3.2. Solubility Properties

The experimental results obtained in this study allow the five halogenated benzaldehydes to be ordered in terms of water solubility (at 25 °C) as follows:

4-Chlorobenzaldehyde > 4-Bromobenzaldehyde > 2,6-Dichlorobenzaldehyde > 2,3-Dichlorobenzaldehyde ~ 2,4-Dichlorobenzaldehyde

For 4-chlorobenzaldehyde, inconsistent solubility values were found in the literature: 919 g/L (20 °C) [36] (6.54 mol/L) and 935 mg/L (20 °C) [37] (0.006652 mol/L). Additionally, several estimated values showed a wide range of discrepancies: 1340 mg/L (25 °C) [38] (0.009532 mol/L), 0.00345 mol/L [ESOL, [39], 0.00815 mol/L [ALI, [39]], 0.00116 mol/L [SILICO-IT, [39]]. These values were obtained from laboratory websites, but the lack of detailed experimental conditions or estimation methods prevents their validation. Nevertheless, the experimental results obtained in this study align with those reported in the Chemical Book [37] and the ones estimates from the ALI method [39]. For 4-bromobenzaldehyde, the literature estimates of solubility include 0.00168 mol/L [ESOL, [39]], 0.00706 mol/L [ALI, [39]], and 0.000634 mol/L [SILICO-IT, [39]]. The experimental result obtained in this study falls between the first and second values reported in the literature. For the other three compounds studied (2,3-, 2,4-, and 2,6- dichlorobenzaldehydes), no experimental solubility data were found in the published literature.

The solubility trends can be explained by considering the effects of halogen substitution on molecular polarity and steric interactions. Halogen atoms, due to their electronegativity, reduce the overall molecular polarity, thereby decreasing solubility. Additionally, *ortho* substitution introduces steric hindrance, which can interfere with the aldehyde group’s ability to interact with water. For 4-chlorobenzaldehyde and 4-bromobenzaldehyde, the *para* position of the halogen minimizes steric hindrance, allowing the aldehyde group to interact more effectively with water. The higher solubility of 4-chlorobenzaldehyde compared to 4-bromobenzaldehyde is attributed to chlorine’s smaller atomic size and higher electronegativity, which enhances its interaction with water molecules.

In contrast, the presence of two chlorine atoms at the *ortho*, *meta*, or *para* positions significantly reduces solubility compared to the monosubstituted compounds. 2,6-Dichlorobenzaldehyde exhibits an interesting behavior: the *ortho* chlorine atoms introduce steric bulk, causing the aldehyde group to twist out of the benzene ring plane. This torsional effect appears to enhance the aldehyde group’s accessibility to water, leading to higher solubility compared to other dichlorinated isomers.

Based on the $\Delta_{hydr}G_{m}^{{}^{\circ}}$ calculated values, the compounds studied can be ordered as follows:

2,3-Dichlorobenzaldehyde ~ 2,4-Dichlorobenzaldehyde > 4-Chlorobenzaldehyde > 4-Bromobenzaldehyde > 2,6-Dichlorobenzaldehyde

For the monohalogenated benzaldehydes studied, the stronger polarizability of the bromine atom enhances interactions with water more effectively, resulting in a more negative values for 4-bromobenzaldehyde compared to 4-chlorobenzaldehyde. In 2,6-dichlorobenzaldehyde, the chlorine atoms in *ortho* positions exert significant polar effects, enhancing the interactions with water despite the steric hindrance. In this compound, the strong electron-withdrawing effect of the *ortho* chlorine atoms increases the electrophilicity of the formyl group, further enhancing the interactions with water and resulting in the most negative value. In practical terms, a highly negative value often indicates stronger interactions with the aqueous environment, which can influence the compound’s mobility and behavior in natural systems such as soils, rivers, and groundwater.

Henry’s law constants reflect the compounds’ tendencies to volatilize from aqueous phases. The observed trend is:

2,4-Dichlorobenzaldehyde ~ 2,3-Dichlorobenzaldehyde > 4-Chlorobenzaldehyde > 4-Bromobenzaldehyde > 2,6-Dichlorobenzaldehyde

Compounds with higher Henry’s law constants, like 2,3- and 2,4-dichlorobenzaldehyde, are more prone to volatilization from water surfaces, contributing to their atmospheric transport. Conversely, compounds with lower constants, such as 2,6-dichlorobenzaldehyde, are less volatile (reduced atmospheric transport) and more likely to remain in the water phase (greater persistence in aquatic environments), potentially influencing their environmental fate and mobility.

### 3.3. Estimation Methods

The values of standard Gibbs energies of sublimation, vaporization, hydration, melting temperatures, water solubilities, and Henry’s constants determined in this study were also used to extend a previously published database, which served for developing Equations (8) to (15) to estimate the properties of benzenes substituted only by halogen atoms (F, Cl, Br, I) [16]. In these equations, *n* is the number of each halogen atom present in the halogenated benzene under study. We decided to extend the estimation of these properties to other non-ionizable benzene derivatives, such as benzaldehydes, which did not show significant intramolecular interactions in the previous studies [16]. With the exception of the equations estimating water solubility of crystalline compounds and Gibbs energies of sublimation (which require knowledge of the melting temperature), all other equations rely only on the number, type, and contributions of the halogen atoms in the benzene molecule. Melting temperature is typically required to estimate mobility properties of crystalline chemicals. While determining this property experimentally is usually straightforward, obtaining accurate predictions for the melting temperature of crystalline substances remains challenging [17]. Monte and Almeida developed Equation (8) to estimate the melting temperature with a standard error of ± 21 K, using only the number of each halogen atom and the logarithm of the symmetry number (*S*) of the halogenated benzene molecule [17]. The symmetry numbers of the compounds studied are 2 for 4-chlorobenzaldehyde, 4-bromobenzaldehyde and 2,6-dichlorobenzaldehyde, and 1 for 2,3- and 2,4-dichlorobenzaldehydes. If the melting temperature is not experimentally known, it can be estimated using Equation (8), though this introduces additional uncertainty into the calculated values of related properties such as $\Delta_{\mathrm{cr}}^{g}G_{m}^{{}^{\circ}}$ and *S*_cr_.

(8) $$T_{m}/K=194+n_{F}\cdot 2.1+n_{Cl}\cdot 29.7+n_{Br}\cdot 48.2+n_{I}\cdot 526+88.8\;{log}_{10}S$$

(9) $$\Delta_{cr}^{g}G_{m}^{{}^{\circ}}(298.15\;K)/kJ\cdot {mol}^{-1}=-11.5+0.056\cdot \left(T_{m}/K\right)-n_{F}\cdot 0.06+n_{Cl}\cdot 4.6+n_{Br}\cdot 7.2+n_{I}\cdot 10.7$$

(10) $${\Delta_{l}^{g}G_{m}^{{}^{\circ}}(298.15\;K)/kJ\cdot {mol}^{-1}=5.1+n}_{F}\cdot 0.09+n_{Cl}\cdot 4.9+n_{Br}\cdot 7.5+n_{I}\cdot 11.2$$

(11) $$\left\{{log}_{10}(p_{cr}/Pa)\pm 0.08\right\}=7.01+n_{F}\cdot 1.0\times 10^{-2}-n_{Cl}\cdot 0.806-n_{Br}\cdot 1.26-n_{I}\cdot 1.87-9.8\times 10^{-3}(T_{m}/K)$$

(12) $$\left\{{log}_{10}(p_{l}/Pa)\pm 0.10\right\}=4.11-n_{F}\cdot 1.6\times 10^{-2}-n_{Cl}\cdot 0.858-n_{Br}\cdot 1.31-n_{I}\cdot 1.96$$

(13) $${log}_{10}\left(S_{cr}/mol\cdot m^{-3}\right)=4.22-n_{F}\cdot 0.163-n_{Cl}\cdot 0.656-n_{Br}\cdot 0.919-n_{I}\cdot 1.28-9.6\times 10^{-3}(T_{m}/K)$$

(14) $$\Delta_{hydr}G_{m}^{{}^{\circ}}/kJ\cdot {mol}^{-1}=-4.3+n_{F}-n_{Cl}\cdot 0.689-n_{Br}\cdot 2.10-n_{I}\cdot 3.20$$

(15) $${log}_{10}(K_{H}^{pc}/{kPa\cdot m}^{3}{\cdot mol}^{-1})\pm 0.10=-0.35+n_{F}\cdot 0.158-n_{Cl}\cdot 0.121-n_{Br}\cdot 0.367-n_{I}\cdot 0.561$$

By using these equations and the experimental results obtained in this study, the average contributions of the formyl group (-CHO) to the aforementioned properties were estimated and are presented in Table 7.

This table plays an important role in extending the previously developed estimation models to a broader range of benzene derivatives. By incorporating the contributions of the formyl group (-CHO), the applicability of Equations (8)–(15) is expanded beyond halogen-substituted benzenes to other non-ionizable benzene derivatives, such as benzaldehydes. The ability to estimate the thermodynamic properties reported in Table 7 using these equations is particularly valuable when experimental data are unavailable due to decomposition, high costs, or limitations in existing experimental methodologies. These contributions are especially important for predicting the behavior of compounds with an environmental impact, particularly those that are hazardous.

## 4. Materials and Methods

### 4.1. Compounds and Purity Analysis

The five benzaldehydes being studied were purchased from Sigma-Aldrich and then purified through sublimation under reduced pressure prior to the experimental measurements. Gas–liquid chromatography (GC) was employed to verify the purity of both the original compounds and the purified samples. An Agilent 8860 GC System chromatograph was used, featuring a 30 m long, non-polar HP-5 semi-capillary column, with an inner diameter of 0.32 mm and a film thickness of 0.25 mm. The stationary phase is composed of (5%-phenyl)-methylpolysiloxane, capable of reaching a maximum temperature of 598 K, and nitrogen was used as the mobile phase with a flow rate of 1.0 cm^3^·min^−1^. The GC system is connected to a flame-ionization detector (FID), which operates with a hydrogen flow rate of 30.0 cm^3^·min^−1^ and a compressed air flow rate of 400 cm^3^/min, using nitrogen as the carrier gas. Additional details about the compounds under investigation, including their purities, and methods of analysis, are provided in Table 8.

### 4.2. Thermal Analysis

#### 4.2.1. Melting Temperatures and Enthalpies

The purified samples of the five compounds were analyzed for their melting onset temperatures and enthalpies using a Netzsch heat flux differential scanning calorimeter (DSC, model 204 F1 Phoenix). It was confirmed that no phase transitions occurred in their crystalline patterns. This apparatus works with a τ-sensor and an intra-cooler system, capable of operating within a 193–873 K temperature range. The sensor is standard for most DSC applications, delivering excellent sensitivity and high resolution simultaneously. It offers outstanding calorimetric sensitivity with extremely short signal time constants, allowing for the effective separation of overlapping thermal effects. For each compound four individual experiments were carried out, with samples placed in hermetically sealed aluminum crucibles. The samples were scanned at a rate of 2.0 K·min^−1^, from 298 K up to 15 to 20 K above their melting temperature, and subjected to two heating-cooling cycles. Controlled nitrogen fluxes were used as both a purge gas (40 cm^3^·min^−1^) and a protective gas (20 cm^3^·min^−1^). The calorimeter’s temperature and heat flow scales were calibrated using four substances from Netzsch’s kit (NETZSCH2022 [40] as well as other high-purity organic reference substances proposed by Sabbah and El Watik [41], Sabbah et al. [42], Della Gatta et al. [43], Roux et al. [44] as well as Chang and Bestul [45]. The individual onset melting temperatures (*T*_m_), along with the related molar enthalpies and entropies for the five compounds, are presented in Table S1, alongside available literature data.

#### 4.2.2. Crystalline Heat Capacities

The crystalline molar heat capacity measurements were conducted following Netzsch’s protocols and recommendations, adhering to ASTM E1269-24 [46], DIN 51 007 [47], and ISO 11357-4 [48] standards. Each measurement required three runs: a blank run, a calibration run (using sapphire), and a sample run. The temperature program included an initial isothermal step at the starting temperature for 25 min, a temperature ramp at 10 K·min^−1^, and a final isothermal step at the finishing temperature for 25 min. Nitrogen was used as the purge and protection gas, with flow rates of 30 cm^3^·min^−1^ and 50 cm^3^·min^−1^, respectively. To verify the reliability of the experimental approach, molar heat capacity measurements were performed using benzoic acid (NIST, 0.99996) and synthetic sapphire (α-Al_2_O_3_, 0.9999) as reference materials within the temperature range of 281–370 K. The relative percentage error of less than 2% compared to the literature values [22] confirms the accuracy of the calibration measurements. All samples were placed in aluminum crucibles with lids (unsealed) and weighed with a precision of ±0.1 μg.

### 4.3. Volatility Study

A previously described and tested static apparatus [49] was used to measure the vapor pressures of the condensed phases (both crystalline and liquid) of the five compounds studied. This installation employs MKS capacitance diaphragm absolute gauges, operating at self-regulated constant temperatures. Our lab is equipped with three pressure gauges. Gauge I, a Baratron 631A01TBEH (MKS instruments, Andover, MA, USA) (*T*_gauge_ = 423 K), measures pressures in the range of 0.5 to 1.3 × 10^2^ Pa and operates within a temperature range of 253 to 413 K. Gauge II, the instrument used in this study, is a Baratron 631A11TBFP (MKS instruments, Andover, MA, USA) (*T*_gauge_ = 473 K), capable of measuring pressures from 0.5 to 1.3 × 10^3^ Pa within a temperature range of 253 to 433 K and gauge III, a Baratron 631D02T8FP (MKS instruments, Andover, MA, USA) (*T*_gauge_ = 473 K), designed to measure pressures between 0.5 Pa and 2.6 × 10^2^ Pa, also within a temperature range of 253 to 433 K. The temperatures of the samples were measured using a Pt100 platinum resistance thermometer, calibrated by comparison with a standard platinum resistance thermometer (SPRT: Tinsley Precision Instruments Ltd, London, England). To prevent vapor condensation, the tubing connecting the cell containing the condensed sample to the pressure gauge is maintained at a temperature higher than that of the sample but lower than that of the gauge. The uncertainty in temperature measurements is estimated to be *u*(*T*/K) = ±0.01, and the expanded uncertainties (0.95 confidence level, *k* = 2) in pressure measurements are given by the expression *U*(*p*/Pa) = 0.1 + 0.0050 (*p*/Pa) for the gauge used. Samples were thoroughly degassed before starting the vapor pressure measurements, ensuring consistent readings at specific temperatures and eliminating any residual volatile substances, including water.

### 4.4. Solubility Experiments

The equilibrium solubility of the five halobenzaldehydes was determined in water using the saturation shake flask method at atmospheric pressure and 25 °C. An excess amount of each compound was added to a known volume of water in triplicate. These concentrated suspensions were continuously shaken (90 rpm) in an OLS Aqua Pro shaking water bath (±0.1 K, Grant Instruments, Cambridgeshire, UK), until thermodynamic equilibrium was achieved, which was determined to be after an average of 72 h. After stirring, the solid phase was allowed to settle for 2 h. The pH of the saturated solutions was measured using a Mettler Toledo (Greifensee, Switzerland) *FiveEasy F20* pH Meter calibrated with standard aqueous solutions (pH 7.00 and 4.01 or 10.1). Those solutions were then filtered through a 0.22 μm syringe hydrophilic filter (13 mm, PTFE, Labfil, Zhejiang, China) and diluted to the required concentration. The concentration of the compounds, expressed in molarity, was measured by absorbances using a Cary-60 UV–vis spectrophotometer (Agilent Technologies, Santa Clara, CA, USA) at 298.15 K (single cell Peltier accessory, ±0.15 K). The results reported in Table 6 are averaged from at least three replicates. Calibration was performed using solutions of known concentrations of each substance in water. These solutions were prepared by adding a precise mass of the substance and a specific volume of the solvent to a flask, then mixing until the substance was fully dissolved. The absorbance of these solutions was measured, and calibration curves were created. These curves were linear, with correlation coefficients better than 0.999.

The accuracy of the experimental setup was validated by comparing the solubility data for benzoic acid in water determined in this work with the mean literature values taken from reference [50], showing deviations of no more than 0.84%.

## 5. Conclusions

This study quantified several key physicochemical properties of 4-chlorobenzaldehyde, 4-bromobenzaldehyde, 2,3-dichlorobenzaldehyde, 2,4-dichlorobenzaldehyde, and 2,6-dichlorobenzaldehyde, providing valuable insights into their behaviors and potential applications. The principal findings are summarized as follows:

- Melting Properties: The melting temperatures and molar enthalpies for all five compounds, as well as their crystalline isobaric molar heat capacities, were determined using differential scanning calorimetry.
- Vapor Pressure Analysis: Vapor pressure measurements conducted across a range of temperatures allowed for the determination of molar enthalpies, entropies, and Gibbs energies of sublimation and vaporization. Corresponding phase diagrams, including the (*p*, *T*) coordinates for the triple points, were also obtained.
- Volatility Assessment: Sublimation vapor pressure measurements rank the volatilities as: 4-Chlorobenzaldehyde > 4-Bromobenzaldehyde > 2,4-Dichlorobenzaldehyde ~ 2,3-Dichlorobenzaldehyde > 2,6-Dichlorobenzaldehyde. These differences are mainly influenced by molecular weight, intermolecular interactions, and crystal packing. The higher volatility of 4-chlorobenzaldehyde is due to its weaker intermolecular forces and lower molecular weight, while stronger interactions in 2,6-dichlorobenzaldehyde reduce its volatility.
- Aqueous Solubility Measurements: The water solubilities of the five halogenated benzaldehydes follow the order: 4-Chlorobenzaldehyde > 4-Bromobenzaldehyde > 2,6-Dichlorobenzaldehyde > 2,3-Dichlorobenzaldehyde ~ 2,4-Dichlorobenzaldehyde. These differences were influenced by the position and number of halogen substituents, affecting molecular polarity and steric hindrance. The higher solubility of 4-chlorobenzaldehyde was due to chlorine’s smaller size and greater electronegativity, while the lower solubility of dichlorinated isomers resulted from increased steric hindrance and reduced polarity.
- Gibbs Energy and Henry’s Constants: By combining volatility and solubility data, the Gibbs energy of hydration and Henry’s law constant were calculated for each compound. The Henry’s law constant indicated that 2,3- and 2,4-dichlorobenzaldehydes had the highest potential for volatilization from water, while 2,6-dichlorobenzaldehyde had the lowest tendency to escape into the atmosphere
- Formyl Group Contribution: Using an estimation model, the contributions of the formyl group (-CHO) to key physicochemical properties of the substituted benzenes were estimated.

Overall, this research deepens the understanding of the selected halogenated benzaldehydes and provides essential data for future studies on their environmental impact and industrial applications.

## Figures and Tables

**Table 1:** Vapor pressure results ^a^.

*T*/K	*p*/Pa	100Δ*p*/*p* ^b^	*T*/K	*p*/Pa	100Δ*p*/*p* ^b^	*T*/K	*p*/Pa	100Δ*p*/*p* ^b^
4-Chlorobenzaldehyde								
*Crystalline phase*								
267.45	0.57	0.4	285.16	4.49	0.1	303.05	28.05	0.4
269.33	0.72	0.5	287.22	5.65	0.7	305.04	34.26	1.4
271.42	0.93	0.5	289.23	6.93	−0.3	306.98	40.71	0.5
273.29	1.15	−0.7	291.18	8.49	−0.5	309.01	49.06	0.3
275.39	1.48	−0.3	293.07	10.45	0.7	310.96	58.06	−0.6
277.25	1.84	−0.3	295.14	12.75	−0.5	312.92	69.28	−0.6
279.32	2.33	−0.4	297.14	15.67	0.0	314.91	82.74	−0.6
281.20	2.90	0.2	299.12	19.07	0.0			
283.26	3.60	−1.2	300.99	23.01	0.4			
*Liquid phase* ^c^								
305.70	48.17 ^d^	0.3	325.71	183.0	−0.4	343.52	520.0	0.3
307.91	56.22 ^d^	−0.1	327.66	206.8	−0.2	345.50	578.0	0.2
309.86	65.09 ^d^	0.8	329.70	235.5	0.4	347.47	640.9	0.0
311.92	75.03 ^d^	0.7	331.69	264.4	0.1	347.50	641.0	−0.1
313.90	86.06 ^d^	0.8	333.66	298.2	0.5	349.45	711.8	0.1
315.83	96.33 ^d^	−0.9	335.64	336.0	0.9	351.43	788.7	0.0
317.92	110.0 ^d^	−1.5	337.61	375.5	0.7	353.30	864.6	−0.4
319.87	125.4	−1.1	339.57	415.3	−0.3	355.30	958.0	−0.2
321.85	143.8	−0.2	339.57	416.0	−0.1	357.33	1058	−0.4
323.75	162.2	−0.2	341.56	468.0	0.6			
4-Bromobenzaldehyde								
*Crystalline phase*								
283.09	0.83	−0.3	298.95	4.98	−0.6	314.77	24.88	0.4
285.23	1.07	−0.4	301.06	6.27	0.0	316.79	29.92	−0.4
287.05	1.33	0.1	302.93	7.60	−0.3	318.78	36.33	0.4
289.17	1.71	0.7	304.86	9.21	−0.9	320.66	43.34	0.7
291.17	2.13	−0.1	306.87	11.43	0.2	322.62	51.66	0.4
292.95	2.63	1.0	308.76	13.82	0.2	324.68	61.49	−0.8
295.00	3.25	−0.5	310.84	17.02	0.5			
297.08	4.12	0.5	312.78	20.28	−0.9			
*Liquid phase* ^c^								
316.76	39.76 ^d^	−1.0	336.48	148.9	−0.1	356.26	463.6	−0.4
318.77	46.26 ^d^	−0.2	338.43	168.4	0.2	358.21	513.4	−0.6
320.73	53.22 ^d^	0.1	340.46	189.9	0.0	360.13	573.1	0.3
322.70	60.73 ^d^	−0.2	342.44	213.5	−0.1	362.14	628.6	−0.8
324.68	69.79 ^d^	0.2	344.41	240.4	0.2	364.12	701.8	0.1
326.64	79.36 ^d^	0.0	346.34	266.7	−0.6	366.10	775.8	0.1
328.63	90.64 ^d^	0.1	348.34	300.1	−0.2	368.08	860.4	0.6
330.60	103.9	1.0	350.32	333.2	−0.9	370.05	946.6	0.5
332.51	117.8	1.3	352.34	375.3	−0.2	371.99	1026	−0.8
334.45	131.9	0.4	354.27	418.5	0.1	373.98	1147	0.8
2,3-Dichlorobenzaldehyde								
*Crystalline phase*								
284.21	0.49	−0.7	302.18	3.78	−1.2	319.59	21.97	−0.5
286.16	0.63	0.9	303.89	4.58	−0.2	321.86	27.35	0.0
288.13	0.78	−1.2	305.78	5.63	0.7	323.56	31.92	−0.4
290.09	0.99	−0.3	308.08	7.12	0.4	325.70	39.35	0.9
292.06	1.26	1.0	309.86	8.45	−0.7	327.48	45.52	−0.7
294.04	1.58	1.1	312.03	10.58	0.0	329.63	56.32	1.3
296.22	2.00	0.2	313.92	12.78	0.1	331.62	65.87	−0.6
298.17	2.47	−0.3	315.60	15.22	1.1			
299.97	3.02	0.2	317.96	18.67	−1.3			
*Liquid phase* ^c^								
327.59	54.84 ^d^	−1.1	347.44	189.1	0.6	367.35	569.4	0.0
329.66	62.05 ^d^	0.4	349.41	214.0	−0.2	369.27	630.4	−0.4
331.60	71.02 ^d^	−0.2	351.50	240.6	0.1	371.17	695.2	−0.6
333.65	80.84 ^d^	0.3	353.48	267.2	0.9	373.23	767.3	−0.2
335.54	91.33	0.3	355.36	297.6	0.7	375.13	843.4	−0.3
337.55	103.4	0.6	357.43	335.9	0.0	377.17	930.5	−0.2
339.54	117.4	0.4	359.33	371.6	0.3	379.07	1011	0.6
341.49	133.6	−0.4	361.39	419.6	−0.8	381.03	1104	1.0
343.48	150.6	−0.3	363.27	461.9	−0.4			
345.45	169.9	−0.5	365.34	516.4	−0.6			
2,4-Dichlorobenzaldehyde								
*Crystalline phase*								
285.78	0.64	0.8	305.23	5.65	−0.3	327.66	50.25	0.1
287.68	0.80	0.3	307.12	6.86	−0.6	329.75	60.90	0.6
289.73	1.02	0.4	308.88	8.29	0.2	331.73	72.78	0.9
291.36	1.23	0.1	310.95	10.12	−0.8	333.66	85.89	0.6
293.15	1.50	−0.6	313.23	12.78	−0.3	335.72	102.0	0.1
294.83	1.81	−0.9	315.25	15.58	−0.4	337.64	119.1	−0.7
296.85	2.29	0.0	317.80	20.05	0.1	339.73	141.9	−0.8
298.81	2.85	0.2	320.56	26.18	0.4	341.66	166.2	−1.0
301.19	3.67	−0.4	322.93	32.84	0.9			
303.16	4.55	−0.1	325.24	40.77	1.2			
*Liquid phase* ^c^								
324.34	57.72 ^d^	−0.2	342.29 ^d^	184.1	0.6	359.46	479.4	−1.0
326.40	66.54 ^d^	0.1	344.36	207.6	0.2	361.34	533.0	−0.4
328.22	75.25 ^d^	0.2	346.33	233.6	0.4	363.54	603.2	0.5
330.22	85.67 ^d^	−0.1	348.33	261.6	0.1	365.63	668.2	0.0
332.21	97.72 ^d^	0.1	349.66	282.0	0.0	367.44	733.6	0.1
334.17	110.6 ^d^	−0.1	351.41	310.8	−0.2	369.34	810.6	0.5
336.16	125.6 ^d^	0.0	353.17	342.8	−0.3	371.29	885.4	−0.3
338.15	141.4 ^d^	−0.5	355.39	390.3	0.4	373.62	998.4	0.3
340.44	163.3 ^d^	−0.2	357.59	437.4	−0.1	375.74	1100	−0.2
2,6-Dichlorobenzaldehyde								
*Crystalline phase*								
292.72	0.50	−0.3	308.53	3.07	0.4	324.42	15.66	0.4
294.67	0.63	−0.6	310.48	3.74	−0.8	326.34	18.87	0.5
296.72	0.81	0.3	312.49	4.61	−1.2	328.34	22.67	−0.2
298.56	1.00	−0.1	314.41	5.68	−0.4	330.30	27.33	0.0
300.60	1.28	1.1	316.45	7.02	−0.2	332.29	32.89	0.0
302.53	1.58	0.3	318.40	8.62	0.4	334.27	39.51	0.2
304.58	1.97	−0.6	320.45	10.52	−0.2	336.23	46.91	−0.4
306.53	2.49	1.3	322.40	12.77	−0.2	338.20	56.28	0.2
*Liquid phase* ^c^								
334.28	48.38 ^d^	0.6	352.10	151.3	0.5	369.87	415.4	0.9
335.87	53.45 ^d^	−0.2	354.09	168.0	−0.9	371.82	456.8	0.1
338.22	62.48 ^d^	−0.3	356.04	190.2	0.0	373.82	506.8	0.0
340.16	71.24 ^d^	0.0	358.03	212.4	−0.5	375.77	559.6	−0.1
342.24	81.73 ^d^	0.3	359.98	239.9	0.4	377.79	620.3	−0.1
344.17	91.46	−0.8	361.98	266.2	−0.5	379.71	682.3	−0.2
346.13	103.7	0.0	363.92	297.7	−0.1	381.71	755.4	0.0
348.09	118.4	0.2	365.92	333.0	0.1	383.62	827.1	−0.2
350.07	133.6	0.3	367.87	372.6	0.7			

^a^ The standard uncertainty of the temperature is *u*(*T*/K) = 0.01 and the expanded uncertainties (0.95 confidence level, *k* = 2) of the vapor pressures are *U*(*p*/Pa) = 0.1 + 0.0050 (*p*/Pa). ^b^ Δ*p* = *p* − *p*_calc_, where *p*_calc_ is calculated from the Clarke and Glew Equation (1) with the parameters shown in Table 2. ^c^ Including supercooled liquid. ^d^ Vapor pressures of the supercooled liquid.

**Table 2:** Standard (*p*° = 10^5^ Pa) thermodynamic properties of sublimation and vaporization of the compounds studied.

Δ*T*	*θ*	$\Delta_{\mathrm{cd}}^{g}G_{m}^{{}^{\circ}}(\theta )$	*p* (Equation (1))	$\Delta_{\mathrm{cd}}^{g}H_{m}^{{}^{\circ}}(\theta )$	$\Delta_{\mathrm{cd}}^{g}S_{m}^{{}^{\circ}}(\theta )$ (Equation (2))	$-\Delta_{\mathrm{cd}}^{g}C_{p,m}^{{}^{\circ}}(\theta )$	*σ*_r_ ^a^
K	K	kJ·mol^−1^	Pa	kJ·mol^−1^	J·K^−1^·mol^−1^	J·K^−1^·mol^−1^	
4-Chlorobenzaldehyde							
*Crystalline phase*							
267.45 to 314.91	298.15	21.47 ± 0.01	17.3	73.3 ± 0.2	173.8 ± 0.7	32.1 ± 9.5 ^b^	0.0062
	291.18 ^c^	22.68 ± 0.01	8.54	73.6 ± 0.1			
	319.43 ^d^	17.79 ± 0.02	123	72.6 ± 0.6			
*Liquid phase* ^e^							
305.70 to 357.33	298.15	20.34 ± 0.03	27.3	56.7 ± 0.6	122.0 ± 2.0	71.2 ± 9.6 ^b^	0.0053
	331.52 ^c^	16.39 ± 0.01	262	54.4 ± 0.1			
	319.43 ^d^	17.79 ± 0.02	123	55.2 ± 0.2			
4-Bromobenzaldehyde							
*Crystalline phase*							
283.09 to 324.68	298.15	24.76 ± 0.01	4.59	79.4 ± 0.2	183.3 ± 0.7	36.9 ± 13.5 ^b^	0.0058
	303.88 ^c^	23.71 ± 0.01	8.40	79.2 ± 0.2			
	330.02 ^d^	18.98 ± 0.03	99.1	78.2 ± 0.7			
*Liquid phase* ^e^							
316.76 to 373.98	298.15	22.94 ± 0.05	9.57	61.2 ± 0.7	128.3 ± 2.4	79.6 ± 7.9 ^b^	0.0057
	345.37 ^c^	17.16 ± 0.01	254	57.4 ± 0.1			
	330.02 ^d^	18.98 ± 0.03	99.1	58.6 ± 0.3			
2,3-Dichlorobenzaldehyde							
*Crystalline phase*							
284.21 to 331.62	298.15	26.30 ± 0.01	2.47	81.2 ± 0.3	184.1 ± 1.0	28.7 ± 14.0 ^b^	0.0081
	307.92 ^c^	24.50 ± 0.01	6.98	81.0 ± 0.2			
	334.82 ^d^	19.60 ± 0.03	87.6	80.2 ± 0.4			
*Liquid phase* ^e^							
327.59 to 381.03	298.15	24.18 ± 0.08	5.81	62.7 ± 1.1	129.2 ± 3.7	71.8 ± 9.6 ^b^	0.0056
	354.31 ^c^	17.29 ± 0.01	282	58.6 ± 0.1			
	334.82 ^d^	19.60 ± 0.03	87.6	60.0 ± 0.4			
2,4-Dichlorobenzaldehyde							
*Crystalline phase*							
285.78 to 341.66	298.15	26.13 ± 0.01	2.64	81.5 ± 0.2	185.7 ± 0.7	35.4 ± 7.7 ^b^	0.0063
	313.72 ^c^	23.25 ± 0.01	13.5	81.0 ± 0.1			
	343.90 ^d^	17.75 ± 0.02	201	80.0 ± 0.5			
*Liquid phase* ^e^							
324.34 to 375.74	298.15	23.40 ± 0.05	7.95	61.9 ± 0.7	129.1 ± 2.4	74.1 ± 6.8 ^b^	0.0037
	350.04 ^c^	17.02 ± 0.01	289	58.0 ± 0.1			
	343.90 ^d^	17.75 ± 0.02	201	58.5 ± 0.1			
2,6-Dichlorobenzaldehyde							
*Crystalline phase*							
292.72 to 338.20	298.15	28.66 ± 0.01	0.95	85.9 ± 0.4	192.0 ± 1.3	31.7 ± 11.8 ^b^	0.0058
	315.46 ^c^	25.35 ± 0.01	6.35	85.4 ± 0.1			
	342.99 ^d^	20.14 ± 0.02	85.7	84.5 ± 0.7			
*Liquid phase* ^e^							
334.28 to 383.62	298.15	25.95 ± 0.10	2.84	66.2 ± 1.2	135.0 ± 4.0	78.9 ± 9.6 ^b^	0.0045
	358.95 ^c^	18.19 ± 0.01	225	61.4 ± 0.1			
	342.99 ^d^	20.14 ± 0.02	85.7	62.7 ± 0.3			

^a^ *σ*_r_ is the relative standard deviation of the fit, defined as ${\sigma_{r}=\left[\sum_{i=1}^{n}{\left(\ln p-{\ln p}_{calc}\right)}_{i}^{2}/\left(n-m\right)\right]}^{1/2}$. ^b^ Adjustable parameter derived from the fittings of Equation (1) to the (*p*, *T*) data. ^c^ Mean temperature. ^d^ Temperature of the triple point. ^e^ Including supercooled liquid.

**Table 3:** Parameters of Equation (3) of the temperature dependence of the crystalline isobaric molar heat capacities of the compounds studied, determined using DSC.

	4-Chloro Benzaldehyde	4-Bromo Benzaldehyde	2,3-Dichloro Benzaldehyde	2,4-Dichloro Benzaldehyde	2,6-Dichloro Benzaldehyde
*a*	87.963	47.890	141.52	97.926	79.188
*b*	−0.068938	0.33078	−0.32612	0.032624	0.13591
*c*	1.0454 × 10^−3^	1.7586 × 10^−4^	1.3994 × 10^−3^	7.4315 × 10^−4^	6.6381 × 10^−4^
*R*^2^ ^a^	0.9996	0.9996	0.9997	0.9996	0.9995
*σ* ^b^	0.20	0.21	0.17	0.22	0.24
Temperature range/K	221.3–292.1	221.3–299.6	241.4–309.6	241.4–314.6	241.4–312.1

^a^ Coefficient of determination; ^b^ Standard error of estimate.

**Table 4:** Values of molar heat capacities of the five compounds studied, at 298.15 K. All values in J·K^−1^·mol^−1^.

4-Chloro Benzaldehyde	4-Bromo Benzaldehyde	2,3-Dichloro Benzaldehyde	2,4-Dichloro Benzaldehyde	2,6-Dichloro Benzaldehyde
$C_{p,m}^{{}^{\circ}}(g)$ ^a^				
127.9 ± 3.8	129.7 ± 3.9	143.9 ± 4.3	144.1 ± 4.3	144.2 ± 4.3
$C_{p,m}^{{}^{\circ}}(cr)$				
Experimental (DSC, this work) ^b^				
160.3 ± 4.1	162.1 ± 2.8	168.7 ± 5.1	173.7 ± 1.9	178.7 ± 1.7
Estimated ^c^				
151.5 ± 17.0	154.2 ± 17.0	170.7 ± 17.0	170.7 ± 17.0	170.7 ± 17.0
$\Delta_{\mathrm{cr}}^{g}C_{p,m}^{{}^{\circ}}$				
$C_{p,m}^{{}^{\circ}}(g)-C_{p,m}^{{}^{\circ}}(cr)$				
−32.4 ± 5.6	−32.4 ± 4.8	−24.8 ± 6.7	−29.6 ± 4.7	−34.5 ± 4.6
−23.6 ± 17.4	−24.5 ± 17.4	−26.8 ± 17.5	−26.6 ± 17.5	−26.5 ± 17.5
Experimental (Equation (1)) ^d^				
−32.1 ± 9.5	−36.9 ± 13.5	−28.7 ± 14.0	−35.4 ± 7.7	−31.7 ± 11.8

^a^ Calculated by means of the Gaussian 09 software package using the vibrational frequencies from G4 calculations [18], scaled by a factor of 0.965 [20]; standard uncertainties were estimated as $u[C_{p,m}^{{}^{\circ}}(g)]=0.03\cdot C_{p,m}^{{}^{\circ}}(g)$ [21]. ^b^ The experimental procedure was tested using benzoic acid (NIST, 0.99996) and synthetic sapphire (α-Al_2_O_3_, 0.9999, NETZSCH), in the temperature range *T*/K = 281–370. The calibration measurements presented a relative percentage error of less than 2% in comparison to the ones described in the literature [22]. ^c^ Acree and Chickos [19]. ^d^ Adjustable parameter derived from the fittings of Equation (1) to the crystalline (*p*, *T*) data.

**Table 5:** Melting properties: temperature, molar enthalpy, and entropy of the compounds studied.

*T*_tp_	*T*_melting_ ^a^	$\Delta_{\mathrm{cr}}^{l}H_{m}^{{}^{\circ}}(T)$ ^b^	$\Delta_{\mathrm{cr}}^{l}S_{m}^{{}^{\circ}}(T)$ ^b^	Method ^c^/(Ref.)
K	K	kJ·mol^−1^	J·K^−1^·mol^−1^	
4-Chlorobenzaldehyde				
	319.85 ± 0.92	18.05 ± 0.80 ^a^	56.4 ± 2.5	DSC/this work
319.43		17.4 ± 0.62		VP/this work
	319.85			[23]
4-Bromobenzaldehyde				
	330.52 ± 0.93	18.79 ± 0.76 ^a^	56.9 ± 2.3	DSC/this work
330.02		19.6 ± 0.8		VP/this work
	334.2	22.6		[24]
	329.15			[25]
2,3-Dichlorobenzaldehyde				
	334.66 ± 0.92	20.24 ± 0.79 ^a^	60.5 ± 2.4	DSC/this work
334.82		20.2 ± 0.9		VP/this work
2,4-Dichlorobenzaldehyde				
	344.11 ± 0.92	21.95 ± 0.76 ^a^	63.8 ± 2.2	DSC/this work
343.90		21.4 ± 0.5		VP/this work
	347.2	20.47		[7]
	345			[26]
2,6-Dichlorobenzaldehyde				
	343.11 ± 0.93	21.50 ± 0.78 ^a^	62.7 ± 2.3	DSC/this work
342.99		21.8 ± 0.75		VP/this work
	343			[27]

^a^ Standard uncertainty calculated through the RSS method combining the expanded uncertainties of the four experimental runs with the standard uncertainties of the DSC calibration *u*(*T*/K) = 0.39 and *u*($\Delta_{\mathrm{cr}}^{l}H_{m}^{{}^{\circ}}(T_{\mathrm{melt}})$/kJ·mol^−1^) = 0.32. ^b^ *T* represents the melting temperature or the temperature of the triple point (*T*_tp_). ^c^ Methods used in this study. DSC: differential scanning calorimetry; VP: vapor pressure measurements.

**Table 6:** Experimental aqueous solubility results at 25 °C, calculated values of Gibbs energy of hydration $\Delta_{hydr}G_{m}^{{}^{\circ}}$, and Henry’s law volatility constants, $K_{H}^{pc}$, for the five compounds studied.

Property	4-Chloro Benzaldehyde	4-Bromo Benzaldehyde	2,3-Dichloro Benzaldehyde	2,4-Dichloro Benzaldehyde	2,6-Dichloro Benzaldehyde
*S*/mol·L^−1^	(8.832 ± 0.035) × 10^−3^	(3.713 ± 0.045) × 10^−3^	(0.8529 ± 0.0035) × 10^−3^	(0.8209 ± 0.0070) × 10^−3^	(1.128 ± 0.0028) × 10^−3^
$\Delta_{hydr}G_{m}^{{}^{\circ}}$/kJ·mol^−1^	−17.7	−18.8	−16.7	−16.5	−19.8
$\log_{10}(K_{H}^{pc}/\mathrm{kPa}\cdot m^{3}\cdot {\mathrm{mol}}^{-1}$	−2.71	−2.90	−2.53	−2.50	−3.07
$K_{H}^{pc}\times 10^{3}/\mathrm{kPa}\cdot m^{3}\cdot {\mathrm{mol}}^{-1})$	1.95	1.26	2.95	3.16	0.851

**Table 7:** Contributions of -CHO group to property Y.

Property (Y)	Contribution of -CHO Group for Property Y	Equation n°
*T*_m_/K	73.2 ± 12.5	(Equation (8))
$\Delta_{\mathrm{cr}}^{g}G_{m}^{{}^{\circ}}$/kJ·mol^−1^	10.4 ± 0.9	(Equation (9))
$\Delta_{l}^{g}G_{m}^{{}^{\circ}}$/kJ·mol^−1^	9.9 ± 1.0	(Equation (10))
log_10_(*p*_cr_/Pa)	−1.82 ± 0.17	(Equation (11))
log_10_(*p*_l_/Pa)	−1.77 ± 0.21	(Equation (12))
log_10_(*S*_cr_/mol·m^−3^ )	0.37 ± 0.09	(Equation (13))
$\Delta_{hydr}G_{m}^{{}^{\circ}}$/kJ·mol^−1^	−12.2 ± 1.4	(Equation (14))
$\log_{10}(K_{H}^{pc}/\mathrm{kPa}\cdot m^{3}\cdot {\mathrm{mol}}^{-1})$	−2.15 ± 0.24	(Equation (15))

**Table 8:** Purity and methods of analysis of the five halobenzaldehydes studied.

Compound	CASNR	Minimum Initial Purity ^a^	Final Mass Fraction Purity	Analysis Method ^b^	% Water Content ^c^
4-Chlorobenzaldehyde	104-88-1	0.97	0.9978	GC (FID)	0.02 ± 0.01
4-Bromobenzaldehyde	1122-91-4	0.99	0.9992		0.03 ± 0.01
2,3-Dichlorobenzaldehyde	6334-18-5	0.99	0.9983		0.02 ± 0.01
2,4-Dichlorobenzaldehyde	874-42-0	0.99	0.9991		0.02 ± 0.01
2,6-Dichlorobenzaldehyde	83-38-5	0.99	0.9990		0.04 ± 0.01

^a^ Minimum purity degree announced by the supplier. ^b^ Gas–liquid chromatography; the results are related to a dry basis. ^c^ Determined through Karl Fisher coulometric titration (mass percentage), using a Methrom titration system, which included an 831 KF Coulometer and the reagent Hydranal TM. The error was assigned as the standard deviation of the mean of four independent measurements.

## Data Availability

The data presented in this study are available in the Supplementary Materials.

## Supplementary Materials

The following supporting information can be downloaded at: https://www.mdpi.com/article/10.3390/molecules30071551/s1, Table S1. DSC results: melting temperatures, molar enthalpies and entropies of the benzaldehydes studied in this work. Table S2. pH values of the saturated solutions of the benzaldehydes studied in this work. Figure S1. DSC thermograms of “wet” (residual solid after solubility measurements) and “dry” (purified original sample) 2,4-dichlorobenzaldehyde. Figure S2. XRD patterns of “wet” (residual solid after solubility measurements) and “dry” (purified original sample) 2,4-dichlorobenzaldehyde.
