# Supplementary material for: Exploring the Volatility, Phase Transitions, and Solubility Properties of Five Halogenated Benzaldehydes
Source: Molecules. 2025 Mar 31;30(7):1551. doi: 10.3390/molecules30071551 (PMC11990670; doi:10.3390/molecules30071551)
Supplement: Supplementary file 1 [file molecules-30-01551-s001.zip › molecules-3507341-supplementary.pdf]

SUPPLEMENTARY DATA

Exploring the Volatility, Phase Transitions, and Solubility Properties of five Halogenated Benzaldehydes

Ana R.R.P. Almeida[1]*, Bruno D.A. Pinheiro[1], Gastón P. León[1], Bogdan Postolnyi[2], João P. Araújo[2], Manuel J.S. Monte[1]

[1]Research Centre in Chemistry (CIQUP), Institute of Molecular Sciences (IMS), Department of Chemistry and Biochemistry (DQB), Faculty of Sciences, University of Porto, Rua do Campo Alegre 687, 4169-007 Porto, Portugal.

[2]Institute of Physics for Advanced Materials, Nanotechnology and Photonics (IFIMUP), Department of Physics and Astronomy (DFA), Faculty of Sciences, University of Porto, Rua do Campo Alegre 687, 4169-007 Porto, Portugal.

*Author for correspondence: ana.figueira@fc.up.pt

This Supporting Material file contains the following contents:

- Melting Properties: temperatures, molar enthalpies and entropies of the compounds studied.
- Thermograms and XRD patterns of 2,4-Dichlorobenzaldehyde
- pH values of the saturated solutions of the benzaldehydes studied in this work
- References

- **Melting Properties**

Table S1. DSC results: melting temperatures, molar enthalpies and entropies of the benzaldehydes studied in this work.

Exp.	$T_{melting}$ (onset) / K[a]	$\Delta_{cr}^{l}H_{m}^{0}(T_{melting})$ / kJ·mol^{-1}	$\Delta_{cr}^{l}S_{m}^{0}(T_{melting})$ / J·K^{-1}·mol^{-1}
		4-Chlorobenzaldehyde	
1	319.78	17.76	
2	319.88	18.05	
3	319.82	18.12	
4	319.93	18.30	
Mean	319.85 ± 0.92	18.05 ± 0.80[a]	56.4 ± 2.5
Lit. [1]	319.85		
		4-Bromobenzaldehyde	
1	330.66	18.80	
2	330.41	18.73	
3	330.52	18.90	
4	330.51	18.74	
Mean	330.52 ± 0.93	18.79 ± 0.76[a]	56.9 ± 2.3
Lit. [2]	334.2	22.6	
Lit. [3]	329.15		
		2,3-Dichlorobenzaldehyde	
1	334.71	20.44	
2	334.64	20.04	
3	334.65	20.14	
4	334.62	20.33	
Mean	334.66 ± 0.92	20.24 ± 0.79[a]	60.5 ± 2.4
		2,4-Dichlorobenzaldehyde	
1	343.98	21.90	
2	344.19	21.91	
3	344.13	22.04	
4	344.15	21.94	
Mean	344.11 ± 0.92	21.95 ± 0.76[a]	63.8 ± 2.2
Lit. [4]	347.2	20.47	
Lit. [5]	345		
		2,6-Dichlorobenzaldehyde	
1	343.27	21.49	
2	343.15	21.67	
3	343.05	21.56	
4	342.98	21.29	
Mean	343.11 ± 0.93	21.50 ± 0.78[a]	62.7 ± 2.3
Lit [6]	343		

[a]Standard uncertainty calculated through the RSS method combining the expanded uncertainties of the four experimental runs with the standard uncertainties of the DSC calibration ($u(T/\mathrm{K})$ = 0.39 and $u(\Delta_{cr}^{l}H_{m}^{0}(T_{fus})/\mathrm{kJ.mol^{-1}})$ = 0.32.

Thermograms and XRD patterns of 2,4-Dichlorobenzaldehyde

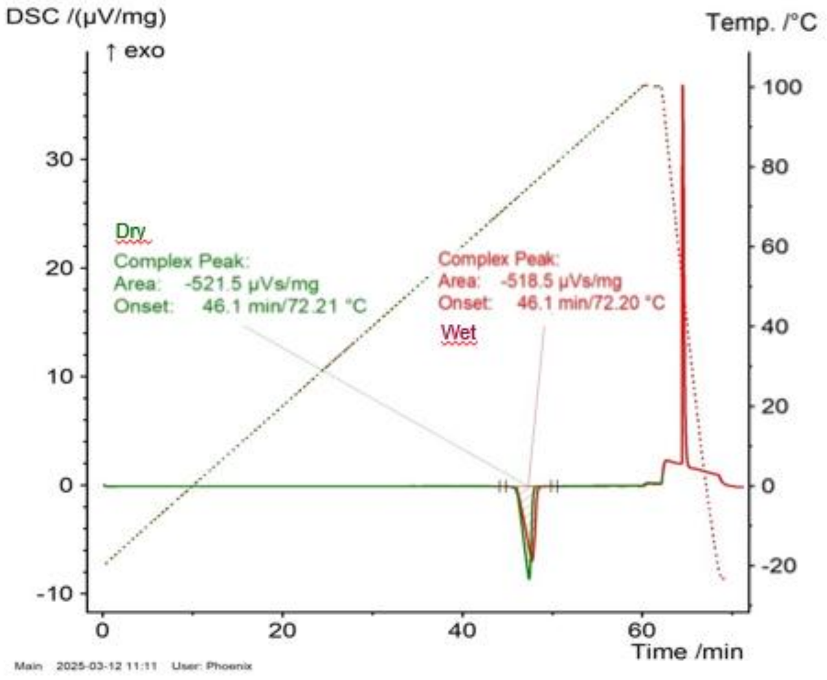

Figure S1. DSC thermograms of "wet" (residual solid after solubility measurements)" and "dry" (purified original sample)" 2,4-dichlorobenzaldehyde.

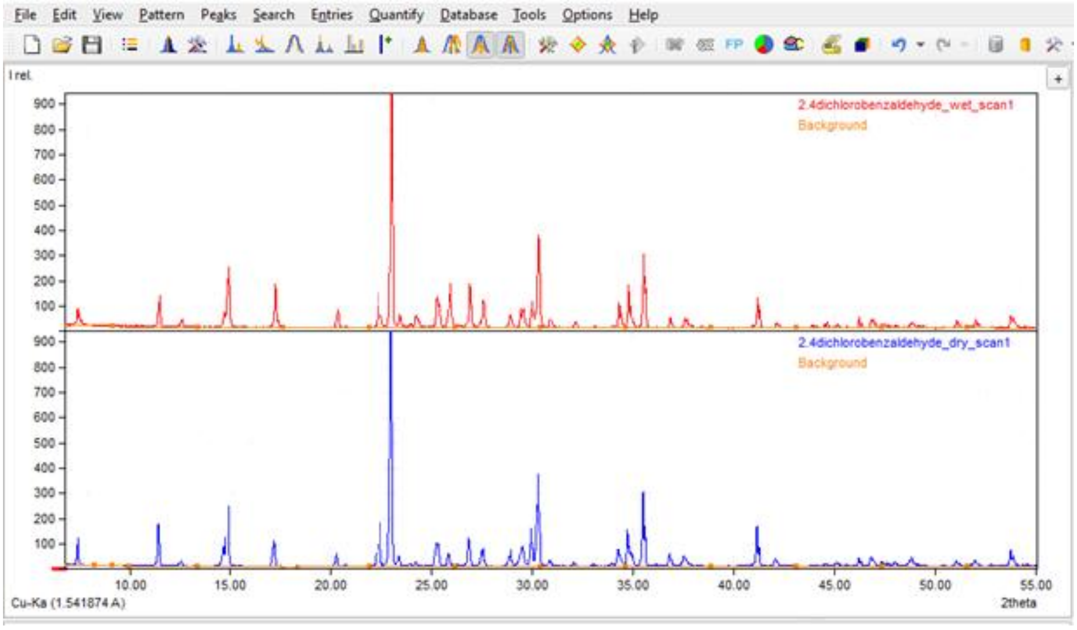

Figure S2. XRD patterns of "wet" (residual solid after solubility measurements)" and "dry" (purified original sample)" 2,4-dichlorobenzaldehyde

Table S2. pH values of the saturated solutions of the benzaldehydes studied in this work.

	4-Chloro benzaldehyde	4-Bromo benzaldehyde	2,3-Dichloro benzaldehyde	2,4-Dichloro benzaldehyde	2,6-Dichloro benzaldehyde
pH	6.61	6.82	6.43	6.57	6.39

References

[1] L. Levi, R.V.V. Nicholls. Formation of Styrene by Pyrolysis of Aromatic Heterocyclic Aldehyde-Aliphatic Acid Anhydride Mixtures on Morden Bentonite, Ind. Eng. Chem. 50 (1958) 1005.

[2] W.E. Acree. Thermodynamic properties of organic compounds: enthalpy of fusion and melting point temperature compilation, Thermochimica Acta 189 (1991) 37-56. https://doi.org/10.1016/0040-6031(91)87098-H

[3] C. Plato, A.R. Jr. Glasgow. Differential scanning calorimetry as a general method for determining the purity and heat of fusion of high-purity organic chemicals. Application to 95 compounds. Anal. Chem. 41 (1969) 330.

[4] S-X. Wang, Z-C. Tan, Y-Y. Di, F. Xu, H-T. Zhang, L-X. Sun, T. Zhang. Heat capacity and thermodynamic properties of 2,4-dichlorobenzaldehyde (C7H4Cl2O). J Chem. Thermodyn. 36 (2004) 393-399. https://doi.org/10.1016/j.jct.2004.01.006

[5] van de Lande. The action of sodium methylate on some derivatives of m-dichlorobenzene. Recl. Trav. Chim. Pays-Bas 51 (1932) 98-113.
